# Video-assisted Thoracoscopic Surgery (VATS) with mini-thoracotomy for the management of pulmonary hydatid cysts

**DOI:** 10.1186/s13019-018-0716-7

**Published:** 2018-05-02

**Authors:** Nizar Abbas, Sarah Zaher Addeen, Fatima Abbas, Tareq Al Saadi, Ibrahem Hanafi, Mahmoud Alkhatib, Tarek Turk, Ahmad Al Khaddour

**Affiliations:** 1 Department of thoracic surgery, Al-Assad University Hospital, Damascus, Syrian Arab Republic; 2 Faculty of Medicine, University of Damascus, Damascus, Syrian Arab Republic; 3 Cardiothoracic surgeon, Damascus University Hospital, Damascus, Syria

**Keywords:** Echinococcosis, Echinococcus granulosus, Hydatid cyst, Video-assisted thoracoscopic surgery (VATS), Mini-thoracotomy

## Abstract

**Background:**

Hydatid cyst is an endemic infectious disease. Various modalities have been provided to approach hydatosis. This article reports a 20-years-experience of a new minimally invasive technique for the management of solitary pulmonary hydatid cysts using video-assisted thoracoscopic surgery (VATS) with mini-thoracotomy.

**Methods:**

We reviewed the medical records of patients who underwent unilateral or bilateral single pulmonary hydatid cyst excision using VATS with mini-thoracotomy. All patients were managed by the same surgeon over the period from January 1996 till January 2015.

**Results:**

The study involved 120 patients aged between 11 and 74 years (median age = 30 years). The overall number of conducted surgeries was 130 (10 patients needed two surgeries). No deaths were reported during or after surgery. No recurrences were seen in the follow-up period that ranged between 10 and 30 months. Three patients (2.3% out of the 130 surgeries) developed post-operative complications: one patient had prolonged air leak and two patients developed empyema.

**Conclusion:**

VATS with mini-thoracotomy is an effective and safe option for managing intact or ruptured solitary pulmonary hydatid cysts. Further studies in controlled prospective design are needed to compare this approach to other modalities of management.

## Background

Hydatid cyst is a parasitic infectious disease, which is endemic in many places around the world, such as the Mediterranean countries, Iran, India, Australia and South America. According to World Health Organization (WHO), the annual incidence of Cystic Echinococcus is up to 220 per 100,000 inhabitants in these countries [1].

The causal parasite of the disease is Echinococcus Granulosus. Humans can serve as intermediate hosts for this organism. It usually infects human organs separately or in groups, especially the liver and the lungs. The hydatid cyst grows slowly and asymptomatically in most of the cases to an extent that some cysts may exceed 20 cm in diameter. This expansive growth can seriously damage the tissue of the hosting organ, and makes spontaneous, traumatic, or intra-operative rupture of the cyst easier. The optimal treatment targets are: complete elimination of the parasite, preservation of the utmost of the healthy tissue, and prevention of recurrence by avoiding the spillage of the cystic fluid and dissemination of the cyst contents [1, 2].

Medical treatment attempts with Benzimidazoles to manage hydatid cysts are countless and persistent. Although this management spares patients the risks of surgery, its efficacy is still limited to specific cases such as hydatid cysts that are smaller than 6 cm, and in inoperable patients, and it is given after surgery to prevent recurrence and secondary echinococcosis [1, 3, 4]. Also, aspiration of cystic fluid yields high risks and small benefits, and is still limited to liver cysts [3, 5].

Surgery with open thoracic surgery, sternotomy or right thoraco-abdominal approach, is the first and the best choice to manage large, multiple or complicated cysts in the lung or the liver [6–8]. Although this surgical approach allows delivering the cysts or removing them along with the damaged tissue without cystic rupture, it is a traumatic and highly invasive procedure for patients [6–8].

Surgical removal using video-assisted thoracoscopic surgery (VATS) has been used to minimize the risks of open surgery. However, it is limited to few clinical entities because of the high risk of postoperative complications such as cyst rupture, cystic fluid spillage and difficulties in controlling bronchial fistulas associated with cysts [9–16].

In this article, we report a series of cases of solitary pulmonary hydatid cyst managed with a new minimally invasive technique using video-assisted thoracoscopic surgery with mini-thoracotomy in order to prevent spillage and facilitate management of the residual cavity and control of the associated bronchial fistulas.

## Methods

### Study design

This is a case-series study. We reviewed the medical records of 120 patients with unilateral or bilateral single pulmonary hydatid cyst, whether it was intact, ruptured, or infected, and those who were managed with thoracoscopic surgery by the same surgeon, who applied the exact same technique for all patients, during the period from January 1996 till January 2015. All consecutive cases which met the inclusion criteria were included. We aimed to investigate operative time, duration of hospital stay, postoperative complications, morbidity, mortality, and recurrence rate.

Exclusion criteria:

1. Big hydatid cysts that are > 15 cm in diameter. Because it might need lung resection.
2. Multiple cysts in a single lung. Thoracotomy provides a better access in these cases.
3. Children under the age of 10 years, due to technical reasons related to anesthetic and thoracoscopic equipment.

### Applied surgical procedure

**Fig. 1:**
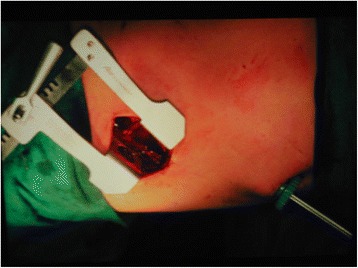
VATS with mini-thoracotomy approach

**Fig. 2:**
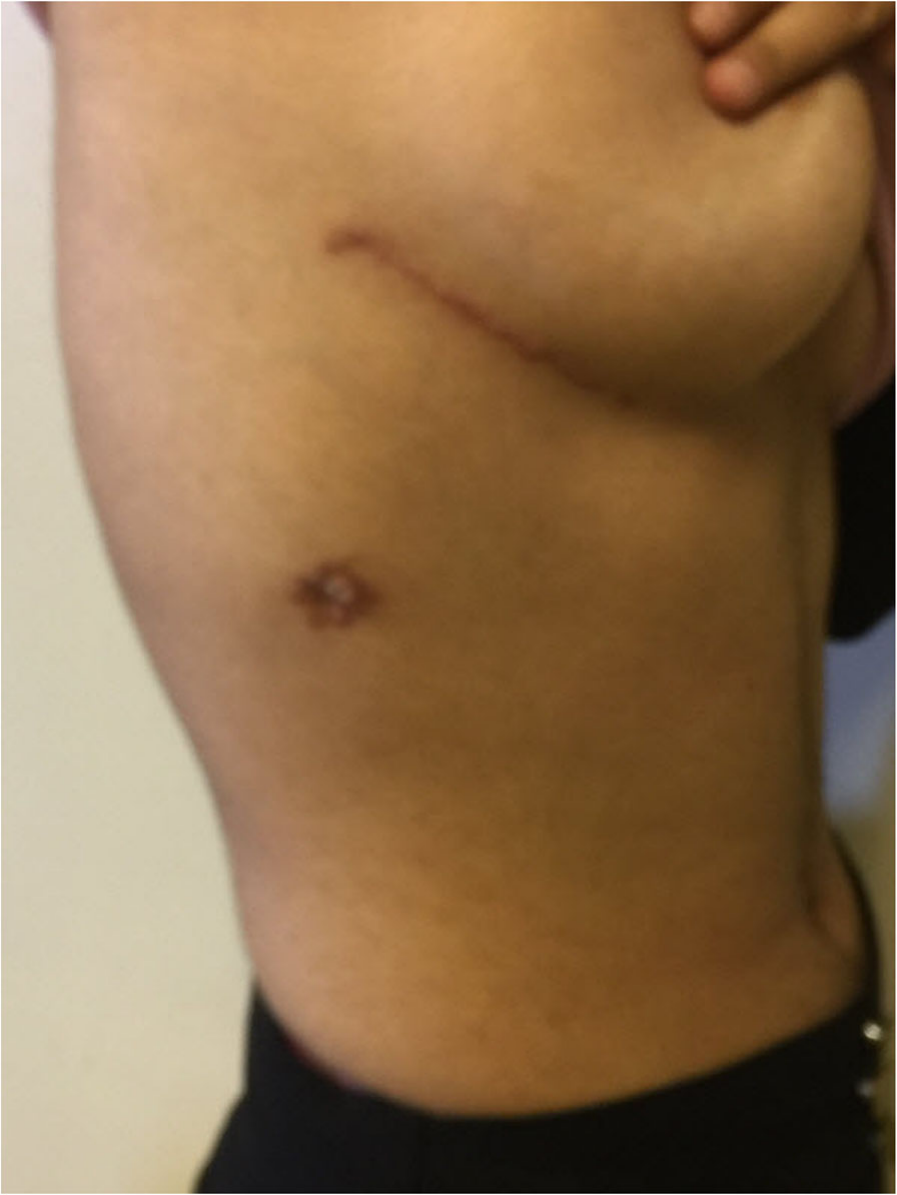
Postoperative cosmetic scar

The surgery is performed with the patient in the lateral decubitus position and under general anesthesia, with the use of double-lumen endotracheal tube in order to isolate the affected lung during surgery. A small entrance for camera is made in the seventh intercostal space at the posterior axillary line, and a small thoracic incision (mini-thoracotomy), with a length of approximately 5 cm, is made directly above the hydatid cyst (location is determined by the use of the scope and the patient’s CT scan images) (Figs. 1 and 2). Then, a small thoracoscopic rib spreader is placed, and pleural adhesions, if present, are partially or completely removed. The pleural cavity is isolated with the use of packs soaked in 20% saline; the lung is pushed away, and the hydatid cyst is pushed closer to the incision area. A lung holder is applied to hold the lung in place.

**Fig. 3:**
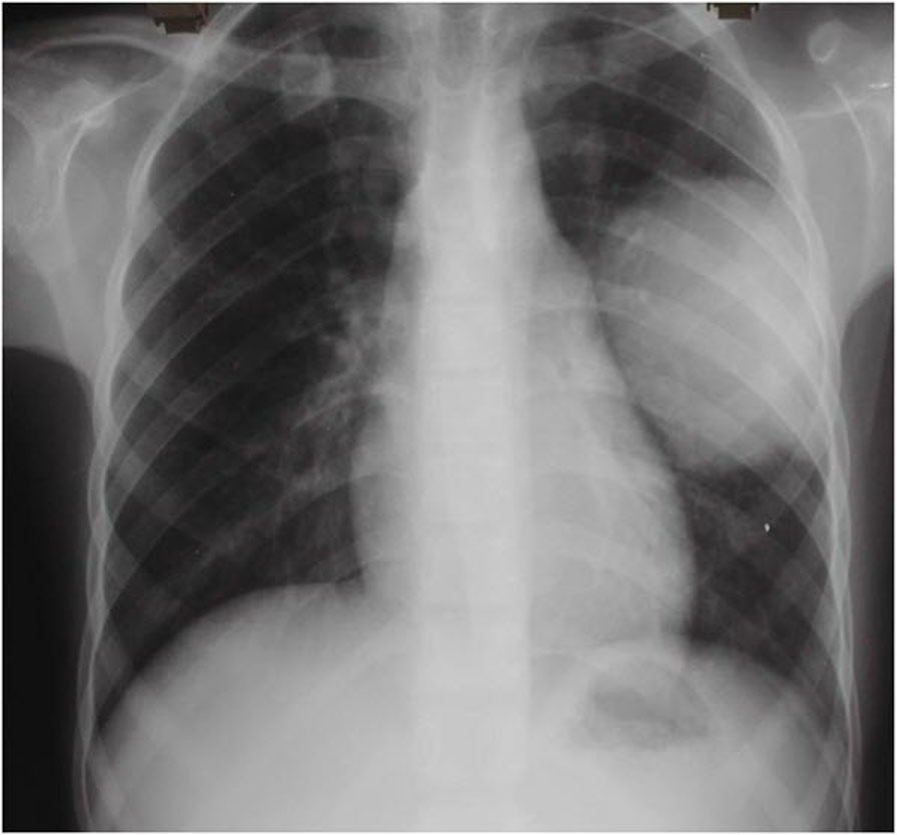
Intact cyst in the left lung

**Fig. 4:**
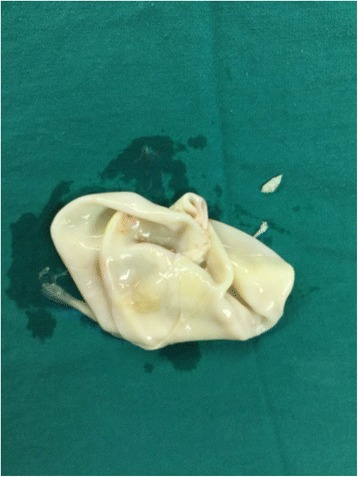
Removed germinal membrane of the cyst

In case of an intact hydatid cyst (Fig. 3), it is aspirated using a 14–16 gauge catheter, which is connected to a 3-ways catheter, which, in turn, is connected to both a syringe filled with scolicidal solution (20% saline), and to an empty bag used to collect the aspirated fluid. Consecutively, some fluid of the hydatid cyst is aspirated and the scolicidal solution is injected. A few minutes later this procedure is repeated several times. Then the fluid is completely aspired, and the catheter is withdrawn. The fibrous capsule is then opened and the germinal membrane is removed (Fig. 4). The remaining cavity is once more sterilized, and the bronchial fistulas are investigated and tightly closed using a 2/0 polyglactine 910; Vicryl. The fibrous capsule is debrided. Finally, the cavity of the hydatid cyst is tightly obliterated by suturing upward from the bottom (capitonnage).

In the event of bilateral hydatid cysts, surgery was performed first on the larger cysts. Preoperative chest radiography and CT scanning were performed on all patients. In patients presented with combined pulmonary and hepatic hydatid cysts, we proceeded with simultaneous combined resection of hydatid cysts in one stage through midsternotomy along with laparotomy or transdiaphragmatic removal of liver cysts in order to avoid three-stage operation of two thoracotomies and a laparotomy.

**Fig. 5:**
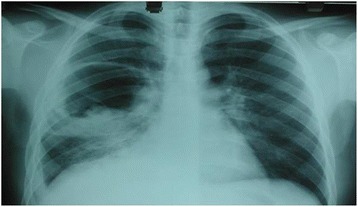
Ruptured cyst

In cases of the ruptured hydatid cysts into the bronchi (Fig. 5) or the pleura, surgery starts by opening the fibrous capsule, followed by aspiration of the remaining fluid then removing the germinal membrane with sterilizing of the remaining cavity, followed by firm closing of the fistulas with the remaining cavity. If signs of any infection are present, the largest portion possible of the fibrous capsule is removed. Definitive diagnosis of infection was considered in the following circumstances: (i) positive culture of bacterial or fungal pathogens from drainage samples, or (ii) surgical or percutaneous drainage of purulent material from the cyst and growth of the organism in blood cultures. Probable diagnosis was defined by surgical or percutaneous drainage of purulent material and one of the following: (i) clinical data of systemic inflammatory response, or (ii) radiological settings suggestive of hydatid cyst infection [17]. Continuous supportive suturing of the cyst margins is applied trying to prevent bleeding and air leak, which lasts for more than the average length of stay or 5 days according to the definition of prolonged air leak (PAL) used in the Society of Thoracic Surgeons database [18]. Cystic cavity is left opened into the pleural cavity.

In all cases, the surgery is ended by repeated washing of the pleural cavity with warm saline, and placing a chest tube for drainage through the same entrance of the camera. Albendazole is administered at a dose of 10 mg/kg continuously for three consecutive months post-surgery.

The follow up was done by chest radiography every 3 months for at least 10 months postoperatively.

## Results

Clinical characteristics of study participants are summarized in Table 1. The study involved 120 patients aged between 11 and 74 years (median age was 30 years). There was no noticeable difference between the proportions of males and females, nor between patients with left- and right-sided cysts. The overall number of performed surgeries was 130; 10 patients had bilateral cysts and required a two-staged surgery for each patient (Table 1).

**Table 1:** Clinical characteristics of the patients

	Number	Percent
Gender		
Males	62	51.7%
Females	58	48.3%
Total	120	
Lung involved		
Left	58	48.3%
Right	52	43.3%
Bilateral	10	8.3%
Clinical type of cysts		
Intact	52	40%
Ruptured into the bronchi	66	50.7%
Ruptured into the pleura	8	6.1%
Infected	4	3.07%
Total	130	

Mean operative time ranged from 75 to 100 min, including the time for anesthesia. Mean hospital stay was 2–3 days for all patients, except for the three patients who developed post-operative complications (2.3% out of the 130 surgeries). Prolonged air leakage (for more than 5 days) occurred in one of these three patients, and it needed an open thoracic surgery to close the bronchial fistulas. The two other patients with post-operative complications had infections: the first one had an empyema that required drainage and regular washing of the pleural cavity with antibiotic therapy according to culture and sensitivity test results, and the other one developed an abscess in the place of the removed cyst and required thoracotomy and lobectomy. The two patients who suffered from infectious postoperative complications had already had infected cysts before surgery.

No mortality was reported during or post-surgery, and no recurrences were seen during the follow-up period (10 to 36 months).

## Discussion

In this case-series, we found that using video-assisted thoracoscopic surgery (VATS) with mini-thoracotomy for the management of a certain group of patients with solitary pulmonary hydatid cysts allowed for total elimination of the parasite, maximum preservation of the healthy tissue, and prevention of recurrences by avoiding the spillage of the cystic fluid.

Published studies in this field are very few and limited to a very small number of cases (Table 2). This is due to the risk of cystic rupture and the spillage of its content during operation, leading to recurrence in the future, and to the difficulty in controlling the accompanied bronchial fistulas and the residual cavity, which increases complication rates and lengthen hospital stay. Such complications may be the reason why some surgeons suggest preserving this technique for dead cysts only [19].

**Table 2:** Results of various studies of VATS for pulmonary hydatid cysts

Authors	Year and country	Patients No.	Age	Mean cyst diameter (cm)	Mean Operative time (min)	Mean Duration of hospital stay (day)	Complication %	Mean period of follow up (month)	Mortality and recurrence
Present study Abbas et al.	Syria 2016	120	Median: 30 Range: 11–74	< 15	75–100	2–3	2.3	10–36	0
Alpay et al. [9]	Turkey 2015	30	Mean: 31.7 Range: 8–85	6.5	102.6	4	13.3	21.8	0
Findikcioglu et al. [12]	Turkey 2012	12	Mean: 32.3 Range: 18–78	(2–7.5)	51.5 (30–100)	2.9 (2–6)	–	29	0
Parelkar et al. [14]	India 2009	3	≤9	< 7	150	4.5	1/3	6	–
Uchikov et al. [16]	Bulgaria 2004	11	Range: 17–55	< 6	–	5	9	6	–
Ettayebi et al. [11]	Morocco 2003	20	Children	–	60	3	–	6–36	0

Our results showed that there was no cystic rupture or cystic fluid spillage during operations, which is supported by the absence of recurrences during the follow up period that lasted for up to 3 years. Moreover, the routine application of prophylactic pharmacological therapy with Albendazole after surgery had helped in preventing recurrence which reached zero in some studies [16, 20]. However, the follow up period in some of these studies was short (about 6 months) and not sufficient to detect all cases of recurrence [11, 14, 16].

The operative time in our study was shorter than that reported in some similar studies [9, 14] by virtue of the operative easiness through mini-thoracotomy. Giving the fact that those studies were limited to small cysts that are less than 7 cm in diameter, which are not usually accompanied by bronchial fistulas, while our study included large cysts that reached 15 cm in diameter.

Duration of hospital stay in our study was relatively short, due to the easiness of control over bronchial fistulas and closure of the residual cavity after cyst removal via mini-thoracotomy, and thus avoiding prolonged post-operative air leakage (a single case out of 130 operations). In some studies [2, 14, 16], where mini-thoracotomy approach was not used, prolonged post-operative air leakage was the most common complication which had led to a longer hospital stay and to an increased complications rate that reached 13.3% in Levent Alpay study [9].

The rate of infectious complications was high in our series, in which we reported two cases: empyema and abscess formation within the place of the removed cyst, in two out of four patients who already had infected cysts. This is consistent with the results of Milind study [15], and requires reconsideration of the role of thoracoscopic surgery in such cases, and looking for a better alternative.

## Conclusion

In conclusion, the case series revealed that (VATS) with mini-thoracotomy is an effective and safe option for managing intact or ruptured solitary pulmonary hydatid cysts. Further studies in controlled prospective design are needed to compare this approach to other modalities of management.
